# Congenital Ventricular Diverticulum or Aneurysm: A Difficult Diagnosis to Make

**DOI:** 10.1155/2018/5839432

**Published:** 2018-11-11

**Authors:** Lloyd Halpern, Carl Garabedian, Neil K. Worrall

**Affiliations:** ^1^Pediatric Anesthesiologist, Shriner's Hospital for Children, 911 W. 5TH Avenue Spokane, Washington 99204, USA; ^2^Pediatric Cardiologist, Providence Sacred Heart Children's Hospital, 101 W. 8th Avenue Spokane, Washington 99204, USA; ^3^Medical Director, Congenital Cardiac Surgery, Providence Sacred Heart Children's Hospital, 101 W. 8th Avenue Spokane, Washington 99204, USA

## Abstract

A pouch protruding from the free wall of the left ventricle may be either a congenital ventricular diverticulum (CVD) or aneurysm (CVA). Being aware of these rare congenital anomalies is critical in making the diagnosis. Differentiating the two is important for treatment decisions. We describe a patient with dextrocardia, Tetralogy of Fallot, and a congenital left ventricular apical diverticulum diagnosed following the induction of anesthesia. CVD and CVA may present in the antenatal period through late adulthood with differing morphology, location, and symptoms. Echocardiography is paramount in the diagnosis and characterization of these lesions. If this anomaly is encountered after the induction of anesthesia or during intraoperative echocardiography, the cardiothoracic anesthesiologist should make the surgical team aware so it can be further characterized and a treatment plan made prior to incision.

## 1. Introduction

A pouch protruding from the free wall of the left ventricle may be either a congenital ventricular diverticulum (CVD) or aneurysm (CVA). Both have distinct presentations, morphology, associated defects, and prognosis. Differentiating the two is important for treatment decisions. We describe a patient with dextrocardia, Tetralogy of Fallot, and a congenital left ventricular apical diverticulum diagnosed following the induction of anesthesia and review the importance of this potentially fatal condition. Written permission was obtained for presentation of this case and patient images.

## 2. Case Presentation

**Figure 1:**
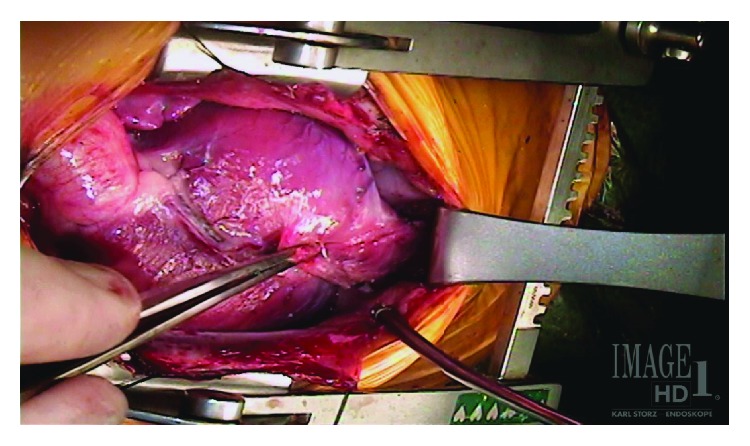
A left ventricular diverticulum extending from the left ventricular apex in a 5-and-1/2-month-old child with dextrocardia and Tetralogy of Fallot.

A male infant was born at 38 weeks of gestation by cesarean section for nonreassuring heart rate and premature rupture of membranes. A prenatal diagnosis of dextrocardia and Tetralogy of Fallot was suspected. Apgars were 9 and 9 at one and five minutes. On physical exam, the child had normal saturations on room air and a 3/6 systolic murmur heard best at the left upper sternal border. Chest X-ray revealed mild cardiomegaly with increased pulmonary vascular markings. Electrocardiogram showed right ventricular hypertrophy. Initial cardiac echocardiography evaluation revealed dextrocardia, Tetralogy of Fallot with subvalvular pulmonary stenosis, anterior malalignment ventricular septal defect (VSD), small muscular VSD, persistent left superior vena cava (LSVC), secundum atrial septal defect (ASD) and normal pulmonary valve morphology. The child did well in the newborn period and was discharged home on no medications with a diagnosis of pink Tetralogy of Fallot. Over the next several months, the child developed signs of heart failure and poor growth and treatment was initiated with diuretics and high-calorie formula. The physical exam was notable for a right-sided point of maximal impulse (PMI), an active precordium that appeared to extend to the subcostal area and an umbilical hernia. Preoperative sedated transthoracic echocardiogram showed mild main pulmonary artery (MPA) and pulmonary valve hypoplasia and suspected left pulmonary artery stenosis (2.5 mm, Z = −3.5). A cardiac catheterization was performed and confirmed the left pulmonary artery stenosis (3.0 mm, Z = −3.0), and a left ventricular angiogram demonstrated dextrocardia and filling of both the aorta and pulmonary artery through the VSD. The child was taken to surgery for repair of Tetralogy of Fallot at 5 1/2 months of age. Following induction of anesthesia, a pulsatile mass was noted in the upper abdomen during line placement. Preoperative transesophageal echocardiography revealed a previously unseen cor triatriatum in the left atrium with flow acceleration above the mitral valve. After median sternotomy, a diverticulum was noted to be extending from the left ventricular apex tracking down the anterior abdominal wall (Figure 1).

**Figure 2:**
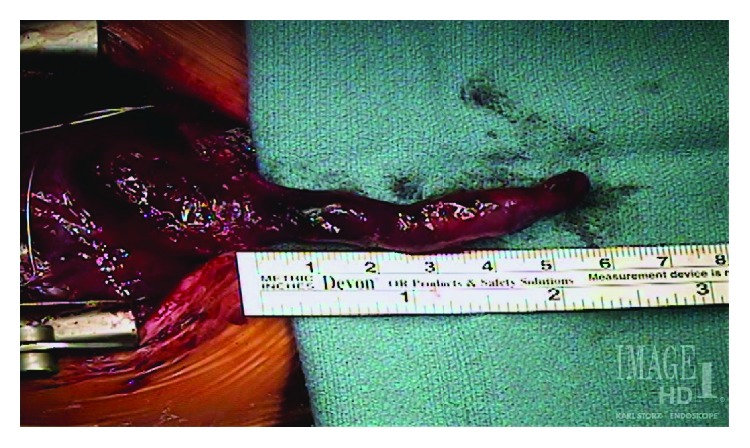
A left ventricular apical diverticulum contracting synchronously with the left ventricle.

Following the initiation of cardiopulmonary bypass the malalignment VSD and additional muscular VSD were closed with a Dacron patch, the right ventricular outflow tract (RVOT) muscles were resected through the pulmonary valve, the ASD closed primarily, and the cor triatriatum resected. After separation from cardiopulmonary bypass the sternal incision was extended 1.5 cm into the upper abdomen. The left ventricular diverticulum was dissected out to the umbilicus and delivered into the chest with gentle traction and revealed a fingerlike pouch contracting synchronously with the left ventricle (Figure 2 and Video 1).

**Figure 3:**
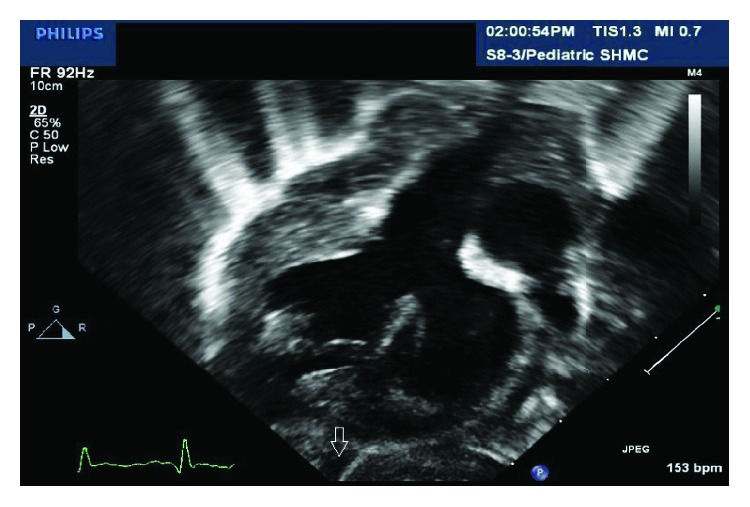
A preoperative transthoracic echocardiography demonstrating a left ventricular apical diverticulum (arrow) not noted at the time of examination.

An echocardiogram probe was placed directly on the diverticulum and demonstrated contractile muscle and blood within the cavity. A clamp was placed across the base of the diverticulum taking care to avoid the left anterior descending artery. Echocardiographic examination confirmed no change in ventricular function and the diverticulum was amputated and the defect oversewn in two layers. The patient recovered uneventfully. Review of the preoperative transthoracic echocardiography revealed a contractile pouch extending from the apex of the left ventricle and contracting synchronously with it (Figure 3). The diverticulum was not visualized on the initial intraoperative transesophageal echocardiography exam.

## 3. Discussion

CVD and CVA are distinct entities with differing histology and presentation [1]. The rarity of the condition has led to inconsistent classification and terminology making diagnosis and treatment decisions difficult. CVD is used to describe an outpouching from a ventricle which contracts synchronously with that chamber and histologically contains all three layers of the ventricular wall (endocardium, myocardium, and pericardium) [2]. CVA is a ventricular protuberance which is akinetic or dyskinetic and on histology is predominantly fibrous tissue with no organized myocardium [3].

The location and morphology of CVA and CVD vary with the age of presentation. CVD presenting in childhood may be divided into those presenting in the apical and nonapical regions of the heart. Those diverticula originating in the apex of the ventricle are always fingerlike contractile pouches extending into the abdomen and are associated with midline thoracoabdominal defects, congenital heart disease, and dextro or mesocardia [4]. They are typically less than 3 cm in length and 1.25 cm in width [5]. They occur much more frequently in the left ventricle but rarely have been reported in the right ventricle or in both ventricles [6, 7]. The midline defect may be obvious (omphalocele and epigastria hernia) or a small rent in the diaphragm discovered at the time of surgery. This collection of findings was described by Cantrell et al. in 1953 [8]. Congenital nonapical diverticulum presenting in childhood are contractile protuberances in multiple shapes and sizes with a narrow or wide connection to the chamber. The size of nonapical diverticulum reported in the literature has ranged from 0.5 cm to as large as 9 cm. They occur most commonly in the left ventricle but have been reported in the right ventricle, biventricular and right atrial position. They are not associated with cardiac or other congenital malformations [4].

CVAs discovered in childhood are akinetic or dyskinetic protrusions extending from the free wall of the heart most commonly present in the left ventricle but may occur rarely in the right. CVAs may vary widely in size and when large compromise ventricular function [9]. They are always associated with a wide connection to the ventricle and are not associated with other congenital anomalies [10]. There may be multiple diverticulum or aneurysms present in a patient but there are only two reports of a patient having both a diverticulum and an aneurysm [11].

Studies reporting CVA and CVD presenting in adulthood describe different morphology and locations than children even when the same classification systems are applied. Fingerlike, contractile diverticulum identical to those presenting in childhood only at the apex of the left ventricle are described in adults in all regions of the left ventricle except the anterobasal and septal area and less than 3% are associated with congenital heart disease. Fifty-six percent of CVD or CVA diagnosed in adults present at the apex of the left ventricle, with nearly one in five being a CVA [12]. The remainder of CVAs presenting in adults are often located in the subaortic region near the aortic valve or in close proximity to the mitral apparatus [7].

There have been 809 published cases of left ventricular diverticulum or aneurysms [11] since the first report in 1816 in Germany [13] and a subsequent report in 1838 in the United Kingdom [14]. Improvements in echocardiography and the introduction of magnetic resonance imaging have brought about a rapid increase in the rate of diagnosis since the 24 known cases prior to 1950 [11]. The first reported surgical resection of a diverticulum was described in 1912 [15]. There have been twenty reported right ventricular apical diverticulum and a single right atrial diverticulum found at autopsy [16, 17]. An autopsy study of 12,924 consecutive children found an incidence of diverticulum or aneurysms of just 0.02% (3 cases) [18]. A retrospective study reviewing 12,271 consecutive adult heart catheterizations found an overall incidence of 0.76% (LVA 0.34% and LVD 0.42%) when the diagnosis of cardiac diverticulum or aneurysm was specifically investigated. Eighty-seven percent were missed at the time of catheterization [12]. A prevalence of 2.2% was reported in 680 consecutive adults undergoing multidetector computer topography angiography for suspected coronary disease [19]. This included diverticulum not extending beyond the free wall of the ventricle, often referred to as clefts, and not considered diverticulum in most classification systems [20]. This increasing prevalence and changing location with advancing age at presentation suggests that diverticulum and aneurysms of the cardiac free wall may develop later in adulthood as a result of increased intrachamber pressure in combination with a congenital predilection. Increased intraluminal pressures twice systemic pressures have been observed in nonapical ventricular diverticulum [21].

In the ante- and neonatal period, CVD and CVA are most often discovered by echocardiography while the child is being evaluated for associated cardiac defects [22, 23]. The most common are Tetralogy of Fallot, venticular septal defect, and tricuspid atresia [4]. The diagnosis may be missed on echocardiography because the left ventricular apex may be difficult to observe and the diverticulum may not align with standard diagnostic views. In infants and children the diagnosis is more often made at the time of cardiac catheterization prior to surgical repair of associated congenital heart defects [24]. In one report, 15% of cases of CVD were first diagnosed when an abnormal abdominal pulsation and small diaphragmatic dehiscence were noted at the time of surgery [4]. The EKG and chest X-ray are typically nondiagnostic in infancy but may show nonspecific anomalies in adults [4, 25]. Cardiac MRI is useful to define the extent of the diverticulum, distinguish it from an akinetic or dyskinetic aneurysms, and for long-term follow-up [20].

Nonapical diverticulum discovered in the neonatal period are most often diagnosed by echocardiography during investigation for arrhythmias. The majority of patients with nonapical diverticulum do not have cardiac dysfunction and may go unnoticed until adulthood [4]. Patients with large CVAs are often identified in an antenatal ultrasound. The prognosis for CVA diagnosed antenatally has generally been reported to be good [26]. However, CVAs may have associated significant ventricular dysfunction. The fetus should be followed with serial ultrasounds as those that continue to enlarge are associated with a poor prognosis [9].

Congenital diverticulum and aneurysms not diagnosed at birth are often asymptomatic and found later in life coincidentally during diagnostic procedures for other indications [15]. The typical age at presentation is 30 to 60 years of age [12, 27]. Common presenting symptoms are angina and atypical chest pain (57%), arrhythmia and syncope (43%), dyspnea (20%), and stroke (6%) [12]. Rupture is rare but has been reported in 33 cases, with 90% of them occurring in patients younger than 18 years [11]. In adult presentation, the diagnosis is made on echocardiography, cardiac MRI, or cardiac catheterization. Patients may present with valvular pathology as those diverticulum and aneurysms in a perivalvular position may be associated with valvular insufficiency, prolapse, or perforation [28, 29].

Congenital left ventricular apical diverticulum when discovered at the time of surgery for associated cardiac anomalies are resected and the chamber wall closed primarily to avoid possible later complications of arrhythmia, embolic stroke, and rupture [30, 31]. The prognosis in these patients from their diverticulum is excellent. The prognosis for nonapical diverticulum diagnosed in the neonatal period is also favorable. The decision to treat is made considering size, symptomatology, and prognosis. Spontaneous regression has been reported in two cases and the overall majority are free of symptoms in early childhood [4]. A nonapical diverticulum has been treated with closure by a PDA device placed percutaneously. That child has done well on follow-up [24]. The feasibility of this treatment depends on the location of the diverticulum and associated symptoms.

The prognosis for CVAs diagnosed in the neonatal period is poor, with a survival rate of 30% reported [4]. Congenital aneurysms diagnosed antenatally may regress or increase in size to consume the ventricle. A reported case of an antenatally diagnosed ventricular aneurysm associated with massive pericardial effusion was treated with en utero drainage of the effusion and the aneurysm reabsorbed prior to birth. Conversely, an aneurysm diagnosed antenatally was followed and sequential ultrasound exams demonstrated continuous enlargement of the aneurysm with en utero demise [9].

Patients presenting in adulthood show an overall benign course. Some authors have recommended surgical excision to avoid later complications [32] and others have recommended a more conservative approach [33]. In a study with a five-year follow-up, none of the 16 patients had died as a result of the lesion [34], and in another report, a patient was followed for 13 years without any change in the diverticulum or symptoms attributable to it [30]. Both diverticulum and aneurysms may be associated with morbidity with nearly 30% of patients experiencing one or more symptoms attributable to the aneurysm or diverticulum. Diverticulum and aneurysms were associated with embolic events in 11% of patients and arrhythmias and syncope in 14% of patients. Of these patients, the majority required ablation, pacemaker, or defibrillator placement [12].

The etiology of congenital apical diverticulum has been postulated to be failure of fusion of the cardiac loop to the yoke sack [8]. Nonapical diverticulum are believed to be the result of failure of normal embryogenesis with a focal defect of the ventricular wall [3]. CVAs may be the result of failure of the formation of normal myocardial tissue secondary to a viral infection or localized ischemia [34, 35].

Congenital left ventricular diverticulum and aneurysms must be differentiated from myocardial clefts and pseudoaneurysms. A myocardial cleft is a contractile, fissurelike protrusion within the myocardium that does not extend beyond the free wall of the ventricle. Clefts contract with systole and are often obliterated in systole. They occur from defects in myocardial fascicle arrangement and are of no prognostic significance. False aneurysms are composed of pericardium which has contained a myocardial rupture and are akinetic. They are typically the result of trauma or infarction [20].

Congenital ventricular diverticulum and aneurysms are rare, potentially fatal anomalies. Being aware of this rare anomaly is critical in making the diagnosis. Differentiating them is imperative because of different presentations, associated disorders, prognosis, and treatment. CVD and CVA may present in the antenatal period through late adulthood with differing morphology and symptoms. Echocardiography is paramount in the diagnosis and characterization of these lesions.

## Supplementary Materials

Supplementary 1 Video 1: a left ventricular apical diverticulum contracting synchronously with the left ventricle.

Supplementary 2 Video 2: preoperative transthoracic echocardiogram demonstrating a congenital apical left ventricular diverticulum contracting synchronously with the left ventricle seen at the bottom of the screen.
